# Go with the flow: de-orphaning focused combinatorial libraries

**DOI:** 10.1186/1758-2946-6-S1-P49

**Published:** 2014-03-11

**Authors:** Michael Reutlinger, Tiago Rodrigues, Petra Schneider, Gisbert Schneider

**Affiliations:** 1 Department of Chemistry and Applied Biosciences, Swiss Federal Institute of Technology (ETH), Zurich, 8093, Switzerland

## 

The fast pace of drug discovery programs, aided by high-throughput screening campaigns, often relies on the generation of combinatorial libraries to identify new chemical entities. The Ugi 4- and 3-component reactions in particular [1], have proven to be robust in producing both tool compounds and drugs [2,3]. Here we report a high-throughput entry into the imidazopyridine scaffold, using a microfluidic-assisted synthesis setup, coupled to a target prediction tool to de-orphan a focused compound library with high success rate, and identify an innovative GPCR-inhibiting chemotype. Combinatorial compounds were correctly identified as ligand-efficient adenosine A_1/2B_, and adrenergic α_1A/B_ inhibitors with *K_i_* values in the low micromolar range.
